# Community knowledge and practice of malaria prevention in Ghindae, Eritrea, a Cross-sectional study

**DOI:** 10.4314/ahs.v23i1.26

**Published:** 2023-03

**Authors:** Amanuel Kidane Andegiorgish, Semhar Goitom, Kidane Mesfun, Michael Hagos, Mussie Tesfaldet, Eyasu Habte, Eyob Azeria, Lingxia Zeng

**Affiliations:** 1 Department of Epidemiology & Biostatistics, School of Public Health, Xi'an Jiaotong University Health Science Center, Xi'an, Shaanxi Province, 710061, China; 2 Ministry of Health, Eritrea; 3 School of Public Health, Asmara College of Health Sciences, Asmara Eritrea

**Keywords:** Malaria, knowledge, practice, prevention, Ghindae, Eritrea

## Abstract

**Background:**

Little is known about community knowledge and practice towards malaria prevention in Ghindae, Eritrea.

**Methodology:**

A community based cross-sectional study design was employed among 380 households. Participants were selected systematically.

**Result:**

More than eight-tenth (86.5%) of the respondents had heard information about malaria preceding the survey; health facilities (54.1%), television (23.7%). Majority (94.2%) mentioned mosquito bite as the main mode of malaria transmission. Fever was the predominantly (89.2%) identified sign/symptoms of malaria. ITN (84.4%) and environmental sanitation (67.3%) were well recognized preventive measures for malaria. Though most households (91%) possess bed nets, but only 37% were ragged on observation. Overall, 64% of the respondents have satisfactory knowledge and 57.3% had adequate practice towards malaria prevention. Malaria knowledge was significantly associated with increased age (p=0.001) and district areas (p=0.022). Malaria prevention practice was significantly associated with Tigrigna and Saho ethnic group (p=0.013), and districts (p=0.02). Districts showed significant difference with an OR=4.56 (95%CI, 1.29-16.09) on knowledge for district 04 and OR=1.98(95%-CI, 1.21-3.26) on practice for district 03 compared to district 01. Knowledge was associated with prevention (OR=1.99, 95%CI, 1.28-3.09).

**Conclusion:**

Overall community knowledge and practice towards malaria prevention were satisfactory. Furthermore, comprehensive community interventions are paramount for effective sustainable control.

## Background

Global efforts for malaria control and eradication have produced a significant reduction in disease incidence. Global malaria incidence decreased from 72% to 59% cases per 1000 population at risk from 2010 to 2017^1^. A reduction in global investment in malaria research and control in 2016 led to an increase of malaria incidence from 211 million cases in 2015 to 216 million cases in 2016^2^ and malaria remains a huge economic burden, particularly in sub-Saharan Africa. In 2017, approximately 451,000 malaria deaths were recorded worldwide, with 90% of the cases and 93% deaths coming from the African region^1^.

Malaria prevention efforts focus on reducing the burden of disease using insecticide-treated mosquito nets (ITNs) particularly long-last insecticidal nets (LLNs) and indoor residual spraying; accurate diagnosis and prompt treatment^3^–^5^.

Several studies have concluded that the successes of malaria control efforts are highly dependent on community knowledge, socio-cultural aspects and early treatment seeking behaviors^6^–^10^. Adequate knowledge of communities on malaria prevention is a key element in designing, executing and meeting the desired outcomes of malaria prevention intervention and studies involving community knowledge and practice have shown that factors including educational level are related to behavior concerning malaria prevention 7, 9. Community mobilization for malaria control and prevention was effective in many countries^11^–^15^, and failure to consider these aspects may impact the achievement of successful sustainable control programs^16^, ^17^. Studies undertaken in Africa, including Eritrea, showed that knowledge of communities regarding malaria was low 18–21. A study by Adhikari and his co-workers has underlined that building a collaborative partnership with local communities would engender a sense of shared responsibility and ownership that would help sustain malaria control and prevention efforts 14, 22.

To achieve the goal of malaria control leading to elimination, the national malaria control program in Eritrea established a primary health care system in which community participants, called community health agents (CHA), work with health committees 22, as is done elsewhere 11. However, knowledge of malaria prevention in Eritrea has not previously been undertaken. The aim of this study was, therefore, to assess the knowledge of communities on malaria prevention practices in an endemic region of the country. The results obtained are expected to complement the existing malaria control strategies undertaken by the national malaria control program.

## Methods

### Study design

A cross-sectional study about knowledge on malaria prevention was conducted in Ghindae Sub-zone, in the Northern Red Sea Zone of Eritrea from 15^th^ March to 14^th^ of April, 2017.

### Study settings

Eritrea is situated in the Horn of Africa and lies north of the equator between latitudes 12°22′ N and 18°02′ N, and longitudes 36°26′21″ E and 43°13′ E. It covers an area of 124,000 sq km. Altitudes range from 0 to >3000 m above sea level. It has a population of 3.6 million with highest densities in the highlands. Temperature ranges from 16 to 45°c with the lowlands being hotter and drier. Eritrea has two rainy seasons from June-September in the central highlands and western lowlands and from October-March in the eastern lowlands along the Red Sea. Four of the six administrative zones in Eritrea are malaria endemic. Malaria is one of the leading public health problems in Eritrea as more than 70% (41 of 58 sub-zones) of the population reside in malarious areas 5,23. Malaria peaks in October in most regions of Eritrea, while March to April is the main transmission season in coastal areas. The shortfall in global funding also affected malaria control interventions in Eritrea, as only 7.6% of (n=156,553) LLINs were delivered in 2016, compared to (n=2,054,194) in 2015 2 and in 2016, Eritrea experienced a 34.1% increase in malaria incidence from 47,300 to 71,800 and more than 70% increase in deaths from less than 100 to 170 compared to 2015 2.

**Figure 1:**
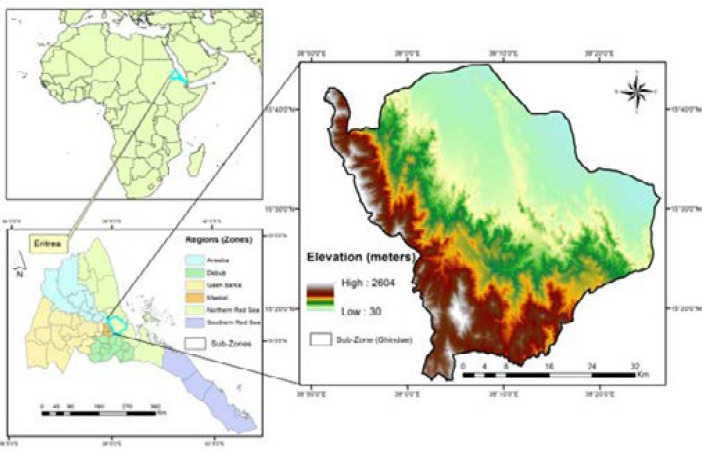
Map of the study area

Ghindae is a subzone of Eritrea in the Northern Red Sea region is the most malaria endemic region in the country^24^ (Fig 1). It is located at an altitude between 0-1000 meters above sea level, in the coastal plain North West of the port of Massawa.

Ghindae town is the capital of the subzone which consists of four districts with an estimated area of 33,861 square kms. Ghindae gets intermittent rains from October to March. The most malarious season is from October to April but occasionally outbreaks occur from June-August.

### Study population

According to the National census a total of 32,917 inhabitants lives in 6,198 households in Ghindae. There are three ethnic groups: Tigre, Tigrigna, and Saho. The main occupations are agriculture, animal husbandry and small-scale businesses.

### Data collection

Data was collected using a structured questionnaire with face-to-face interviews. Originally the questionnaire was prepared in English, and was later translated into the local languages for the interviews. It was translated back to English for consistency. An observation checklist was used to record ITN use during the household visit. A pilot study was conducted in 30 households, two days before the actual data collection, following which; minor amendments were made to the questionnaire. After a brief description of the objectives of the study to participants and after obtaining verbal informed consent, trained public health graduates, with more than three years of working experience, conducted the interviews.

### Sample size calculations and Sampling

Sample size was calculated using a single population proportion formula 25. To the knowledge of the researchers, no information was available regarding the level of knowledge towards malaria prevention in Ghindae. The minimum sample size to estimate a 50% prevalence of knowledge on malaria prevention was used to obtain the desired sample size using the following formula.

n= z2p(1-p)/d2

where n= the desired sample size; z= 1.96 (the Z statistics for 95%CI), p= 0.5 (the proportion of existence of knowledge on malaria prevention), q=1-p, and d= 0.05 (the degree of precision in proportion of one).

The calculated sample size for the desired precision of the confidence interval was 384. Since the population size is small in comparison to the sample size, the population correction factor was introduced

n2= n1 * N/ n1 +N = 362

The corrected sample size became 361. To make this sample stable we added a 5% oversample for the assumptions of non-responders or missing households, and the final sample size equal to 380. Number of households among the 4 districts was calculated proportional to size. Every household (k = 6198/380 ≈16) was selected using a systematic random sampling technique, and one member of the household family (the husband, wife or adult>15years old) was interviewed. The first household was selected using the lottery method from first up to 16th household.

### Data processing and analysis

Data was entered and analyzed using SPSS version 23. Categorical variables were described using frequencies and percentages while continuous variables were described using means and standard deviations and are presented in the form of tables and figures. Binary logistic regression was used to test for possible association of sociodemographic variables and knowledge towards malaria prevention. Crude and adjusted odds ratio along with their 95% confidence intervals were calculated, *p*<0.05 was considered as significant.

### Ethical consideration

Initial ethical approval was obtained from the ethical committee of the Asmara College of Health Sciences. A formal letter attached with a protocol on the objective of this study was presented to the Ministry of Health Department of Research and Human Resources Development. The Human Resources Development office approved and subsequently forwarded it to the Zonal branch of Ministry of Health in Massawa, Eritrea. Subsequently, a letter from the zoba was presented to the Ghindae Administration office and the research team explained verbally the objective of the study. Verbal consent was obtained from each participant prior to commencement of data collection.

### Overall, Knowledge scores

Questions on malaria transmission, common signs and symptoms, mosquito behavior, prevention, treatment and practice towards malaria prevention were asked. Scores were binary ‘one’ being a correct answer and ‘zero’ an incorrect one. For questions with multiple responses, a score was given based on the number of correct choices. An overall knowledge score was calculated by adding up the scores of each respondent across all questions. Using the mean as the cut point participants who scored less than the mean were considered as inadequate and greater than the mean as adequate 20 Similarly, a number of questions were generated that gauge practices on malaria prevention. An overall practices score was determined for each respondent by adding up of the scores across malaria practice questions 20.

## Results

### Socio-demographics characteristics

A total of 379 participants (households) out of 380 participated in this study (i.e., a response of 99.7%). Eighty percent of the Houses were made of stones and bricks while 20% were made of clay and woods. The average age of respondents was 40 (SD 15.3) years. The average family size in surveyed households was 4.93 (±0.12 SE, range 1-12). Three-quarters of the respondents were female. Nearly one-quarter (24%) of the respondents were illiterate. 82.3% were Muslims (Table 1).

**Table 1:** Socio-demographic characteristics of respondents

Variables	Frequency	Percent (%)
Administrative areas		
District 01	175	46.2
District 02	53	14.0
District 03	127	33.5
District 04	24	6.3
Sex of respondent		
Female	286	75.5
Male	93	24.5
Age of respondent		
15–24	49	12.9
25–34	101	26.6
35–44	101	26.6
>45	128	33.8
Religion of respondent		
Muslim	312	82.3
Christian	67	17.7
Ethnic group		
Tigre	230	60.7
Tigrigna	69	18.2
Saho	80	21.1
Educational status		
Illiterate	91	24.0
Elementary	121	31.9
Junior	85	22.5
Secondary	68	17.9
post-secondary	14	3.7

**Figure 2:**
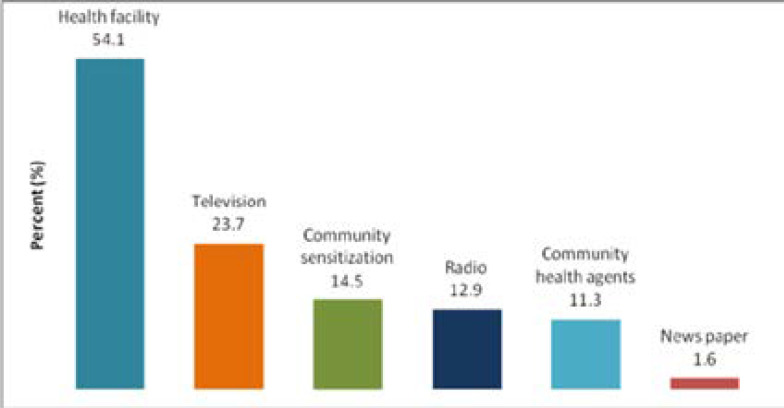
Source of information about malaria

During the transmission season, in the year preceding the survey, 86% of the respondents had heard information about malaria. A health facility (54.1%) was the predominantly mentioned source of information about malaria (Fig.2). The information provided concerned ITN usage (49.3%), environmental sanitation (47.5%) and early treatment seeking (31.1%).

In this study 133 (35.1%) of the participants did not know the CHA in their place of residence.

### Distribution of Knowledge about malaria

Ninety-four percent (n=357) of respondents mentioned mosquito bites as the main mode of transmission of malaria from person to person. Only 2.4% replied that malaria can be transmitted from contaminated water and 0.5% replied that malaria cannot be transmitted from person to person (Table 2).

**Table 2:** Respondent's distribution of knowledge about malaria

Variables	n	Percent (%)
**Malaria is transmitted**		
By the bite of mosquito	357	94.2
Contaminated water	9	2.4
Eating cane of maize or sorghum	1	0.3
Other methods	15	4.0
**Mosquito breeding site**		
Spring water	57	15.0
Sink	58	15.3
Open well	28	7.4
Old tires	57	15.0
Stagnant water, old pot, tank and tins	289	76.5
Other	102	26.9
**Mosquito biting time**		
Night	283	74.7
Both day and night	75	19.8
Day	6	1.6
Don't know	15	4.0
**Methods of Malaria prevention**		
Keeping our environment clean	110	29.9
Clearing of bushes around the house	40	10.4
Use of antibiotics	29	7.7
Use of ITN	320	84.4
Use of anti-malarial prophylaxis	327	86.3

More than three-fourths (76.5%) of the respondents stated that mosquitoes breed in stagnant water including household tanks, old pots and tins. A small percentage (26.9%) stated other mosquito breeding sites like dirty water, waste water, latrines, flowers, and leaves.

Regarding the biting times of mosquitoes, 74.7% of the respondents replied the mosquito that transmits malaria bites during night time, 19.8% during day and night and 1.6%-day time only.

Taking anti-malaria prophylaxis during the malaria seasons (86.3%), sleeping under ITN (84.4%), and environmental sanitation (29.9%) were the most commonly mentioned malaria preventive methods.

Fever was the most frequently mentioned (89.2%) symptom of malaria followed by chills and vomiting as shown in (Table 3).

**Table 3:** Respondent's knowledge about signs and symptoms of malaria

Sign & Symptom	n	Percent (%)
Fever	338	89.2
Chills	212	55.9
Vomit	191	50.4
Joint pain	180	47.5
Bitter taste	113	29.8
back pain	98	25.9
Headache	97	25.6
Sweat	52	13.7

### Association between Socio-demographic factors and knowledge of malaria prevention

Overall, 90% of the participants had knowledge that the entire population is at risk of malaria. Sixty four percent (64%) of the respondents had adequate knowledge towards malaria prevention with scores greater or equal to 10 out of a possible 17 points. The average mean knowledge score was M±SD=10±1.8.

A significant relationship on knowledge of malaria was found with increased age (*p=0.001*) and District area (*p=0.022*) (Table 4). After adjusting for potential confounding variables, the association remained the same, and respondent over 35 years old were 2.6 times more knowledgeable about malaria prevention compared to the age group of 15-24 years (Table 4). Participants from District 04 were four times more knowledgeable about malaria prevention than participants from District 01.

**Table 4:** Association between Sociodemographic factors and knowledge about malaria prevention

Variables	Crude OR (95%CI)	Adjusted OR (95%CI)
**Administrative area**		
District 01	Ref	Ref
District 02	1.3(0.68–2.46)	1.23(0.64–2.37)
District 03	1.25(0.78–2.02)	1.25(0.77–2.02)
District 04	4.7(1.34–16.23) *	4.56(1.29–16.09) *
**Age of respondent**		
15–24	Ref	Ref
25–34	1.59(0.79–3.16)	1.66(0.82–3.33)
35–44	2.95(1.44–5.99) **	2.99(1.46–6.14) **
>45	2.58(1.31–5.07) **	2.55(1.29–5.07) **
**Sex of respondent**		
Female	Ref	
Male	1.39(0.83–2.29)	†
**Religion of respondent**		
Muslim	Ref	
Christian	0.99(0.57–1.72)	†
**Ethnic group**		
Tigre	Ref	
Tigrinya	0.97(0.56–1.69)	†
Saho	0.26(1.37–0.79)	
**Educational status**		
Illiterate	Ref	
Elementary	1.33(0.74–2.39)	†
Junior	0.81(0.44–1.5)	
Secondary	0.77(0.41–1.48)	
Post secondary	0.72(0.23–2.27)	

* Indicates (p <0.05)

** (p<0.001)

† no result are displayed because they were not included at multivariate level

### Availability and utilization of bed net (ITNs)

At least one ITN was available in the majority of the households 91% (n=345). The average number of ITNs per household was 2.6. More than 85% of the households, who had the ITNs, owned it two years before the study.

ITN utilization among children less than five years was 76.2%, and among the groups of 6-20 years was slightly lower (57.8%).

Only 77% of the participants had slept under an ITN the night before the survey. The most abundant reasons mentioned for not using ITN were: shortage of ITNs (28.3%), not having ITNs (7.1%), allergic to ITNs (2.4%) and mosquitoes not present (6.4%). Sixty-five percent of respondents said that they always sleep under an ITN (Table 5). About one third (37%) of the households hang ITNs above the sleeping location on observation.

**Table 5:** Respondent's distribution of practice of malaria prevention

Variables	n	Percent (%)
**Sleep under ITN the night before the study**		
yes	293	77.5
no	85	22.5
**What do you do to prevent from malaria**		
Always sleep under ITNs	247	65.2
Clean my environment	255	67.3
Use chemoprophylaxis	51	13.5
Filling	116	30.6
Clearing the bushes and grass around the house	40	10.6
IRS	8	2.1
Avoid sleep in humid area	379	100
Burn wood and dung in my house	22	5.8
**Bed net utilization outside the house**		
Households with family members sleeping outside	218	57.5
Family members sleep outside and use ITN	160	42.2
**Reasons of not using bed net**		
It causes shortness of breath	12	3.2
No availability of mosquito	20	5.3
Old bed net	11	2.9
Allergy	5	1.3
No bed net	27	7.1
Other reason	36	9.5
**If you get malaria signs and symptoms, what do you do?**		
Go to health facility	369	97.4
Go to community health agent (CHA)	21	5.5
Take traditional medicine	14	3.7
Other	6	1.7
**In the last malaria season environmental sanitation**		
Family was involved in the sanitation activities	266	70.2
House being sprayed using IRS	349	92.1
House being painted after sprayed	25	6.6

*Indicates (p <0.05)

Seventy percent of the respondents participated on all kinds of environmental sanitation in the year preceding the survey and 92% of the houses had been sprayed (Table 5).

### Practice of respondents towards malaria prevention

More than half (57.3%) of the respondents had adequate practices towards malaria prevention getting a score of ≥9. Overall practice score was Median ±1.5 IQR=9±4.5. The most common malaria prevention practice of the respondents was ITN use (65.2%) and environmental sanitation to reduce mosquito breeding sites (67.3%). Besides, a small number of respondents were using protective clothes like linen sheets and electronic insecticide sprayers.

A significant association was found between malaria prevention practices and respondent's ethnic group (*p=0.013*), respondent's district area (*p=0.02*) and among those who have good knowledge about malaria (*p=0.015*). However, age, sex, educational level and religion were not significantly associated with malaria prevention practices. After adjustment, only district area, ethnic group and knowledge were found to be significantly associated with malaria prevention practices (Table 6).

**Table 6:** Associates between socio-demographic factors and practice on malaria prevention

Variables	Crude OR(95%CI)	Adjusted OR(95%CI)
Administrative area		
District 01	Ref	
District 02	2.21(1.16–4.18)*	1.77(0.89–3.49)
District 03	2.13(1.33–3.42)**	1.97(1.19–3.26)**
District 04	2.75(1.08–6.97)*	2.04(0.67–6.22)
Ethnic Group		
Tigre	Ref	
Tigrinya	2.02(1.15–3.59)*	1.79(0.98–3.29)
Saho	1.77(1.04–2.98)*	1.24(0.65–2.36)
Malaria knowledge		
Inadequate	Ref	
Adequate	2.05(1.34–3.14)**	1.99(1.28–3.09)**
Sex of respondent		
female	Ref	
Male	1.18(0.73–1.89)	†
Age of respondent		
15–24	Ref	
25–34	1.35(0,68–2.68)	†
35–44	1.24(0.25–2.46)	
>45	1.10(0.57–2.13)	
Religion of respondent		
Muslim	Ref	
Christian	1.66(0.95–2.9)	†
Educational status		
Illiterate	Ref	
Elementary	1.40(0.81–2.42)	†
Junior	1.69(0.93–3.09)	
Secondary	1.65(0.87–3.12)	
Postsecondary	1.02(0.33–3.15)	

* indicates (p <0.05)

** (p<0.001)

† no result are displayed b/c they were not included at multivariate level

Additionally, sleeping under an ITN the night before the survey was found to be significantly associated with young age (*p<0.001*) and higher educational status (*p=0.042*), whereas sex has no association (*p= 0.792*) (Table not shown).

### Health seeking behavior

Ninety-seven percent of the respondents identified malaria as a serious and potentially fatal disease if left untreated. Because of that 97.4% of the respondents said that they would seek treatment within 24-48 hours from the health facility whenever they felt sick. Besides, 2.6% took homemade herbal medicine like chemor, humor, aloe and would consult the community health agents.

## Discussion

Knowing knowledge of communities about malaria prevention and its source is essential for targeting interventions. Therefore, this study aimed at assessing the level of knowledge towards malaria prevention in the most malaria endemic region in Eritrea 24. Overall, 64% (243 of 379) of the household respondents had adequate knowledge on malaria and 57% (216) had good practices on malaria prevention. Most of the respondents believed that malaria can be prevented by regular use of ITNs (84.4%). However, the practice of regular ITN utilization in this study was only 65%, much lower than other study 9. Consistent with a study from Ethiopia, perceived prevention using anti-malaria prophylaxis was high (86.3%) 26.

In this study, 86.5% of the respondents heard messages about malaria during the transmission season, in the year preceding the survey. This is 60% higher compared to 2012 5, but lower than that obtained in similar studies [26-28]. Differences in socio-demographic characteristics, livelihood, life styles and behavior may have contributed to this difference. More than half of the respondents had heard about malaria from health facilities. Even though, CHAs mobilization was designed to reach the young, low-literacy, or other traditionally hard-to-reach populations 35% of the total respondents did not know the CHAs in their area. Therefore, the roll of CHAs is still sub-optimal in the area and further work is needed to raise their profile. The most common malaria messages received were about ITN use (49.3%), environmental sanitation (47.5%) and early treatment seeking (31.1%). Similar studies cconducted in other settings have demonstrated a comparable proportion on awareness 29. Nevertheless, our finding was lower than Nigerian and South African studies 28, 30.

The majority (94.2%) of respondents identified mosquito bites as the main mode of person-to-person malaria transmission, which is consistent to the 2012 study in Eritrea^5^ and elsewhere 28, 31 but greater than a hospital-based study in Eritrea 21, a district study in Northern and Southern Ethiopia 23 ,20, 26 and three districts in Southern Africa^7^.

Regarding to the early identification of signs and symptoms of malaria, 89.2% recognized fever as the major symptom of malaria in line with a 2012 household study in Eritrea and others 5, 7, 10, 16.

Adequate knowledge on mosquito breeding sites is crucial for isolating risks and taking measures by the community. In this study, stagnant water, old pots, tank and tins were the most predominantly (76.5%) mentioned breeding sites. This is higher compared to studies from Nepal and Ethiopia 20, 29. Similarly, knowing mosquito biting times is very helpful for practicing avoidance measures. Three-quarters of the respondents knew that the mosquito that transmits malaria feeds during the night. This implied that, further work is required since inadequate knowledge on mosquito biting times may lead to enhanced exposure and unsuccessful prevention measures. Almost all (97.4%) participants in the study had a positive attitude towards treatment seeking behavior from a health facility if any family member developed the signs and symptoms suggestive of malaria. This is similar to a study from South Africa in which 99% of the participants sought treatment from a health facility 30. This may be due to the fact that more emphasis has been given to treatment seeking behavior through education and communication in the study site.

Despite the fact that ITNs are dispensed for free in Eritrea, only 91% of the studied households own at least one ITN similar to the proportion in 2012 5, but greater than earlier studies in the country 21, 22. One possible reason for the non-ownership in the present study could be population movement from place to place for pastoralism and trade. However, our findings were much greater than similar East African study 32. Therefore, intervention scaling up on ITN distribution is needed to achieve complete protection of people residing in high malaria risk areas.

ITNs were available for less than three to five household members. An absence of mosquitoes, inadequacy of ITNs and allergic reactions were the reasons mentioned for not using ITN on regular basis. This shows there was a slightly decreased awareness on prevention practices, similar to another study from Tanzania 16. Further work is needed to adhere with the national malaria prevention and control guidelines; every person should sleep under ITN every night throughout the year. These misperceptions may have resulted in the surge of malaria cases and deaths in 2016 in Ghindae 2,24. Nevertheless, ITN use among Ghindae inhabitants was higher than similar communities in South Africa [30]. On observation during the study visit, only 37% of the bed nets had holes.

More than two-third (67.3%) and 70.2% of the household respondents participated in all forms of sanitation activities during the malaria season starting two and one year previous to the present study. Nine-tenth (92%) of all households were sprayed before the study. This entails community involvement on prevention and control of malarial is not yet integral and further work must be required to scale it up and make it sustainable.

Children are particularly vulnerable to malaria and ITN use in children under 5 years showed a steady increase from 58.6% in 2004 through 67% in 2012 to 76.2% in the present study 5, 22. Although high this still left almost a quarter of children without adequate protection. Increased focus on prevention among children remains important.

Participants who had adequate knowledge about malaria were found to show adequate practices on malaria prevention. Knowledge and practice were significantly associated (*p<0.001*). This is concordant with other studies, which showed that the level of knowledge on ITNs was a significant predictor of prevention practice 7, 9, 10, 26. However, other variables like gender, religion, and educational levels were not significantly associated with knowledge and practice on malaria prevention.

Participants from district 02, 03, and 04 had better knowledge of malaria and its prevention methods. This could be due to proximity of the three districts to the ever-flowing river (Mai Adkemom) which passes through district 04, district 02, and district 03 from up to down and which might have resulted an increased awareness of participants towards malaria. Besides, significant arranged malaria control interventions by the local branch of the ministry of health and the community took place along the river districts in accordance with the regular local malaria prevention practices which could have a positive influence on knowledge and prevention practices of the residents.

## Strength and limitation

We have used a systematic random sampling method to ensure the representativeness. Household interview and observational records ensure the accuracy of the information. However, using a cross-sectional study cause and effect associations cannot be investigated.

## Conclusions

Despite concerted efforts of national malaria control, there remained a gap in knowledge and practice of many respondents in the Ghindae community. For effective and sustainable control of malaria, interventions to enhance knowledge and practice of communities on malaria prevention must be instigated. Community interventions should consider the difference on knowledge and practice of respondents in regard to district areas, religion, ethnic group, age and educational levels.

## Data Availability

The dataset generated and analyzed for this study are not publicly available due to participant privacy but are available from the corresponding author on reasonable request.

## Abbreviations

ITN: Insecticide Treated bed Nets
OR: Odd's Ratio
CI: Confidence Interval
